# Deliberate Error-Based Learning in Dental Radiography: An Educational Study

**DOI:** 10.3390/dj14070417

**Published:** 2026-07-08

**Authors:** Andy Wai Kan Yeung, Ray Tanaka, Kuo Feng Hung, Varut Vardhanabhuti, Andrew Nalley

**Affiliations:** 1 Oral and Maxillofacial Radiology, Applied Oral Sciences and Community Dental Care, Faculty of Dentistry, The University of Hong Kong, Hong Kong, China; 2 Department of Diagnostic Radiology, Li Ka Shing Faculty of Medicine, The University of Hong Kong, Hong Kong, China

**Keywords:** error-based learning, error management training, intra-oral radiography, dental education, simulation, radiographic technique errors

## Abstract

**Objectives**: We aimed to explore whether incorporating a deliberate error-based learning activity, adapted from error management training (EMT), could enhance undergraduate dental students’ understanding of common intra-oral radiographic faults and their phantom-head imaging performance. **Methods**: This randomized two-arm educational study involved Year 2 dental undergraduates who completed pre- and post-intervention phantom-head imaging and a short multiple-choice test on radiographic errors. Students were allocated either to a brief, slide-based teaching session on imaging faults (conventional group) or to a hands-on activity in which they intentionally produced and corrected faulty images under supervision (EMT group). Main outcomes included MCQ scores, confidence ratings, overall imaging performance (percentage of maximum possible score), and distribution of diagnostic image quality categories. Two-sample t tests were used to check for inter-group differences in the change in the post–pre numerical scores. The Cochran–Mantel–Haenszel (CMH) linear-by-linear test was used to check for inter-group differences in the change in image distribution by diagnostic quality before and after the intervention. **Results**: Eighty-seven students contributed a total of 735 radiographs. Both groups showed improvements in MCQ scores and confidence within groups. Diagnostic acceptability of student radiographs was already high at baseline and remained so afterwards, with no significant differences between groups in MCQ gains, confidence changes, imaging scores, or shifts in diagnostic image quality distributions. **Conclusions**: Although the single, short EMT-style activity did not outperform conventional teaching on immediate outcomes, the study demonstrates that deliberate error-based learning is feasible in a pre-clinical radiography setting and well-received by students. The findings also help to clarify the circumstances under which EMT is most likely to yield benefits, suggesting that longer or more structured EMT sessions, particularly those involving metacognitive scaffolding or more challenging imaging scenarios, may be needed before performance differences can emerge.

## 1. Introduction

Producing diagnostically acceptable intra-oral radiographs is a key undergraduate competency. Inadequate radiographic technique may result in non-diagnostic images, repeated exposures, unnecessary radiation dose, and delays in diagnosis. Therefore, pre-clinical radiography teaching aims not only to familiarize students with radiographic equipment, image receptors, holders, and exposure geometry, but also to develop their ability to recognize and correct common technical errors such as cone cuts, receptor misplacement, incorrect vertical angulation, incorrect horizontal angulation, elongation, foreshortening, and overlapping contacts.

Traditional radiography teaching commonly emphasizes demonstration of correct technique followed by supervised student practice. While this approach provides procedural clarity and may reduce early learner uncertainty, it may provide limited opportunity for students to explore the causes and consequences of errors. In contrast, error management training (EMT) [1], or productive failure in some texts [2], encourages learners to engage with errors actively and to use mistakes as learning opportunities. This approach has been associated with improved transfer of learning in some educational contexts, particularly when learners are required to adapt their knowledge to new or unfamiliar tasks. From a theoretical perspective, EMT is closely linked to self-regulated learning processes, including error detection, reflection, and strategy adaptation [3]. By encouraging learners to actively engage with errors rather than avoid them, EMT may promote deeper cognitive processing and enhance learners’ ability to transfer knowledge beyond routine tasks [4]. However, the extent to which these mechanisms operate effectively may depend on the nature of the task, the availability of feedback, and the learning environment.

In a famous EMT study by Keith and Frese [1], students were divided into error-avoidant or error management groups and asked to replicate two PowerPoint slides given by the experimenters in a 30 min training phase. The former group was given step-by-step instructions and asked to follow exactly the steps to reach the goal. The latter group was not given such instructions but instead encouraged to make errors and learn from them. Immediately after, they were asked to replicate three PowerPoint slides in a 36 min test phase. Results showed that the error management group produced more accurate slides than the error avoidance group, particularly on the tasks requiring adaptive transfer (new tasks) instead of analogical transfer (familial tasks). It implied that through intentional mistake commitment with a trial-and-error approach, students were more familiar with the PowerPoint program as a whole, as they were more capable of using functions not already covered by the instructions given in the training phase.

A conceptually related study was conducted in radiography, in which students were divided into traditional learning and EMT groups [5]. The former group watched a video (with unspecified duration) that showed a radiographer performing procedures accurately for a patient, and discussed what went well and why. The latter group watched a video (with unspecified duration) that showed a radiographer performing procedures with multiple errors for the same patient, and needed to systematically identify the errors and their implications. After the intervention, the students individually participated in a 30 min scenario during which they needed to carefully interact and handle a patient actor with simulated injury and take diagnostically acceptable radiographs. Results showed that the EMT group had better overall performance and smaller performance variability.

In dental radiology, it is not possible to encourage students to commit errors when taking radiographs for patients, due to ethical concerns and the adherence to the “as low as reasonably achievable” (ALARA) principle [6]. EMT can be applied to simulation-based training without posing risk to actual patients [7]. In particular, phantom-head simulation provides a controlled environment in which students can safely observe, produce, and correct radiographic faults without exposing patients to unnecessary radiation. This creates a potential opportunity to apply deliberate error-based learning in dental radiography education.

Despite the theoretical relevance of EMT to radiographic education, evidence in dental radiology remains limited. It is unclear whether a short, deliberate error-based learning activity can improve students’ conceptual understanding of radiographic faults or their immediate technical performance in phantom-head imaging compared with conventional teaching. Therefore, this study evaluated the effects of a brief EMT-inspired activity on Year 2 dental students in terms of their knowledge level, confidence, and phantom-head radiographic image quality. The use of a phantom head is a common and effective setting for dental radiographic training prior to clinical practicum [8]. The intervention was compared with conventional teaching. It was hypothesized that students who underwent EMT should achieve better knowledge levels and radiographic performances.

## 2. Materials and Methods

### 2.1. Sample Size

This was a parallel-arm randomized educational study embedded within a compulsory radiography session for Year 2 dental undergraduate students. It included the entire Year 2 undergraduate dental cohort enrolled in the compulsory radiography session (*N* = 92). Five students were absent, resulting in a final sample of 87 students who completed all study procedures. As such, no sampling was performed. However, no formal sample size calculation was conducted, and the study may therefore be underpowered to detect small between-group differences, particularly in the presence of ceiling effects. The students had learned radiographic anatomy but not radiographic techniques before coming to this session. This study was approved by the Institutional Review Board of The University of Hong Kong/Hospital Authority Hong Kong West Cluster (HKU/HA HKW IRB, approval no. UW 25-025). Informed consent was obtained from the participating students.

### 2.2. Equipment

The armamentarium details are as follows. The intra-oral X-ray machines used were the Heliodent Plus (Dentsply Sirona, Charlotte, NC, USA) and Phot-X II (Takara Belmont, Osaka, Japan) with a rectangular collimator. The phantom heads used were DXTTR III—natural (Dentsply Sirona, Charlotte, NC, USA). The imaging system used was the Planmeca ProScanner 1.0 and its associated imaging phosphor plates (Planmeca Oy, Helsinki, Finland). The X-ray holders used were Kwik-Bite with index (Kerr Dental, Brea, CA, USA) for bitewing and Rinn XCP (Dentsply Sirona, Charlotte, NC, USA) for periapicals.

### 2.3. Experiment Flow

**Figure 1:**
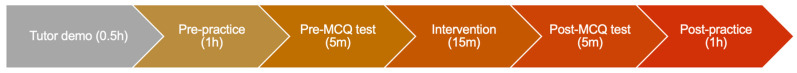
Flow of the radiographic session. This also reflects the allocation of participating groups (“Intervention”) and completion of all study phases without attrition.

Students attended the radiography session in 20 small groups, with each group having 4–6 students. These small groups were randomized in advance to either the conventional teaching group or the EMT group. For pragmatic teaching reasons, all students attending the same small-group session received the same intervention. The sessions were divided into 6 segments (Figure 1):

(1) They started with a 30 min tutor demonstration on how to take a bitewing, an anterior periapical, and a posterior periapical with the paralleling technique.

(2) Pre-practice: Since there were 2 intra-oral X-ray rooms for teaching and learning, the 4–6 students worked in groups of 2 or 3. Each group was instructed to try their best to complete a full-mouth series (15 periapicals + 2 bitewings) in 1 h. They were instructed to take radiographic images according to a color-coded sheet, so that each student could experience as much variety as possible, covering the anterior and posterior regions, as well as the upper and lower arches. Students were encouraged to work independently within their assigned roles, but informal peer discussion within pairs or triads was permitted, reflecting the collaborative nature of clinical training. No formal feedback was provided by tutors during this phase, allowing baseline performance to be assessed without immediate instructional influence.

(3) Pre-MCQ: After the pre-practice, students took a tailor-made 10-question MCQ test. The preset duration was 5 min, but students were allowed to extend the time until test completion. MCQs were used to assess conceptual understanding of radiographic faults in a structured and time-efficient manner. This approach allowed standardized comparison across groups, although it may not fully capture applied diagnostic reasoning.

(4) Students then received a 15 min experimental intervention. The 20 small groups had been randomized into two intervention groups in advance: (a) the conventional group (*n* = 45), with 15 min slide-based teaching on common image faults, and (b) the EMT group (n = 42), with students encouraged to reproduce designated faulty images on a phantom head and then rectify them (including but not limited to incorrect vertical angulation causing elongation/foreshortening, improper horizontal angulation resulting in overlapping contacts, incorrect phosphor plate positioning, cone cut errors). At the end of the 15 min period, they were briefed on the image faults that they failed to produce. All intervention sessions were conducted using standardized instructions delivered by the same tutor to ensure consistency across groups. In other words, each of the 4–6 students who attended the session together were collectively classified into either conventional or EMT group.

(5) Post-MCQ: Students took another 10-question MCQ test for 5 min. The questions and answer options were the same as for the pre-intervention MCQ test, but their orders were shuffled randomly. The number of MCQ items (n = 10) was determined pragmatically to minimize disruption to the teaching session while allowing rapid assessment of key radiographic error concepts immediately before and after the intervention. The same MCQ items were used in the pre- and post-intervention assessments to ensure direct comparability of conceptual understanding. However, this design might have introduced a testing effect and potentially inflated score improvements independent of the intervention. As both groups were exposed to the same assessment approach, any such effect was expected to be non-differential.

(6) Post-practice: Finally, students spent another 1 h completing a second set of full-mouth series. This time, the color-coded sheet demanded students to swap duties and take radiographs for locations not taken by themselves during the first set.

### 2.4. Outcome Measures

For the pre-practice and post-practice with the phantom head, each radiographic image taken by the students was scored by an author (A.Y.) with 10 years of experience in teaching dental radiology, with the following scores given: 0 (diagnostically unacceptable), 1 (diagnostically acceptable), or 2 (excellent with no error). This 3-tier diagnostic quality scoring system was referred to from [9]. To assess intra-examiner reliability, a random subset of 100 radiographs was re-evaluated by the same examiner after a 1-month interval. Weighted Cohen’s kappa was 0.90, indicating very good agreement. Students were allowed to retake faulty radiographs; however, only the original takes were analyzed and the number of retakes was not systematically recorded. As such, the potential influence of retake behavior on image quality distribution cannot be evaluated and should be considered in the interpretation of results. For the pre-MCQ and post-MCQ tests, the total score ranged from 0 to 10, and students needed to rate their confidence level in attempting each of the pre- and post-MCQs on a Likert scale from 1 to 5 (5 being the most confident).

### 2.5. Statistical Analysis

Since each student took a different total number of radiographs, their image quality scores were summated and then expressed as a percentage of the maximum possible score attainable (called phantom-head score hereafter). The changes in the phantom-head score, MCQ score, and MCQ confidence were tested for significance within each group by paired t tests. The changes were computed as [post-intervention—pre-intervention] and then compared between the two intervention groups by 2-sample t tests. To account for potential clustering effects due to group-level randomization, intra-class correlation coefficients (ICCs) were estimated using a one-way random effects model to quantify clustering within small groups. The design effect was calculated as 1 + (m − 1) × ICC, where m represents the average cluster size. Moreover, the change in the distribution of radiographic images among the diagnostic categories between the two intervention groups was tested by the Cochran–Mantel–Haenszel (CMH) linear-by-linear association test. Results were statistically significant if *p* < 0.05.

## 3. Results

**Figure 2:**
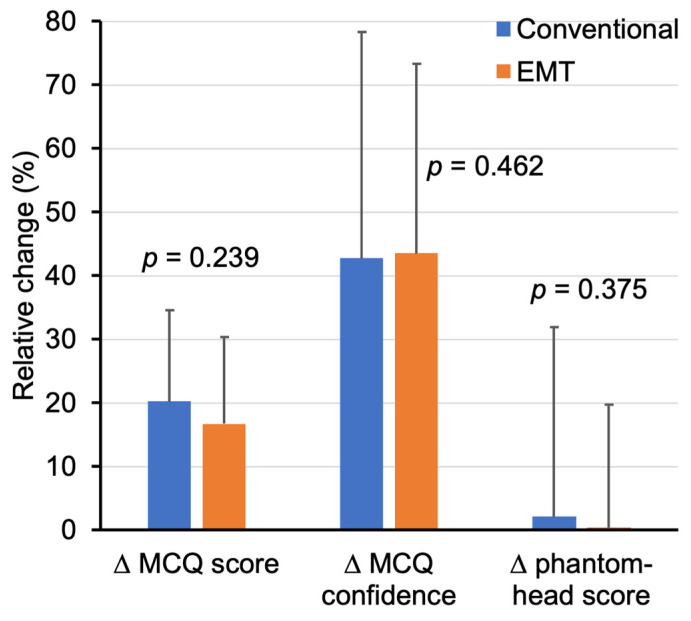
Changes in MCQ score, MCQ confidence, and phantom-head score.

Eighty-seven students completed all study procedures, with 45 allocated to the conventional group and 42 to the EMT group. Across the study, 735 radiographs were analyzed. The conventional group contributed 227 pre-intervention and 161 post-intervention radiographs, while the EMT group contributed 204 pre-intervention and 143 post-intervention radiographs. Table 1 shows that the two groups had similar baseline (pre-intervention) performance in terms of MCQ and phantom-head scores and MCQ confidence (*p* > 0.05). Both conventional and EMT groups significantly improved their mean MCQ score after the 15 min intervention, by approximately 2 points and 1.7 points, respectively (both *p* < 0.001). Both groups had significantly higher confidence in answering the MCQs after the intervention (both *p* < 0.001). Meanwhile, both groups showed mild improvement in their phantom-head scores (conventional group: *p* = 0.632; EMT group: *p* = 0.890). All three metrics showed no significant between-group differences (*p* > 0.05, Figure 2). Effect sizes for between-group differences in change scores were small to negligible. Cohen’s d values were 0.25 (95% CI [−0.17, 0.68]) for MCQ improvement, −0.02 (95% CI [−0.44, 0.40]) for confidence change, and 0.07 (95% CI [−0.35, 0.49]) for phantom-head performance, indicating minimal differences between the two instructional approaches.

The ICC values ranged from 0.00 to 0.25 across outcomes. Moderate clustering was observed for MCQ scores (ICC = 0.25 pre-intervention; 0.08 post-intervention), while confidence ratings showed low to moderate clustering (ICC = 0.08–0.15). In contrast, phantom-head performance demonstrated minimal clustering (ICC ≈ 0.00–0.01). Corresponding design effects ranged from 1.00 to 1.83, indicating a modest reduction in effective sample size due to clustering.

Diagnostic acceptability was high at baseline. In the conventional group, 90.3% of pre-intervention radiographs were acceptable or excellent, while 92.6% of EMT-group pre-intervention radiographs were acceptable or excellent (Table 2). Post-intervention, the acceptable/excellent rates were 93.2% in the conventional group and 92.3% in the EMT group. The distribution of images among the three categories did not show significant between-group differences (pre-intervention: *p* = 0.459; post-intervention: *p* = 0.832). Similarly, the change in the distribution showed no significant between-group difference (*p* = 0.550).

## 4. Discussion

The present study did not demonstrate a significant advantage of deliberate error-based learning over conventional instruction in terms of short-term knowledge gains or phantom-head imaging performance. However, this finding should not be interpreted as evidence against the effectiveness of EMT, but rather as an indication of the specific conditions under which its benefits may not readily emerge in dental radiography training. One important factor is the high baseline performance observed in this cohort, with more than 90% of radiographs already classified as acceptable or excellent prior to intervention. This ceiling effect substantially reduced the sensitivity of the outcome measures and likely limited the ability to detect further improvements. Rather than representing a methodological flaw alone, this finding highlights a key contextual feature of radiography training in which strongly guided techniques and structured workflows may constrain observable performance variation. The >90% acceptable/excellent image rate is consistent with the overall periapical rejection rate of 16.38% reported across the literature [10]. On the other hand, ICC and design effect findings suggested that clustering effects were present for knowledge and confidence outcomes but were minimal for phantom-head performance. Therefore, the lack of between-group differences in radiographic performance is unlikely to be primarily attributable to clustering effects.

Our findings contrast with the seminal EMT study by Keith and Frese [1], which reported clear advantages of EMT over error-avoidant instruction, but specifically on adaptive transfer tasks that required learners to solve new problems rather than repeat familiar ones. In their experiment, students were asked to replicate PowerPoint slides, and before submission they could visually compare their work directly with the “gold standard.” This immediate and transparent feedback allowed learners to readily judge whether their products matched the target and to iteratively refine their outputs. In the present study, however, students faced a very different feedback environment. When taking intra-oral radiographs, learners cannot easily determine in real time whether an exposure will be diagnostically acceptable. The radiographic outcome only becomes apparent after the phosphor plate is scanned and the latent image appears on the computer screen. Although students can externally verify some procedural components such as correct tube-head angulation, alignment of the rectangular collimator, proper assembly of the X-ray holder, and appropriate placement of the phosphor plate, other critical aspects are far less observable.

For example, it can be challenging, or even impossible, to confirm whether the phosphor plate remains fully stabilized without unintended bending, rotation, or “leaning away” from the holder before exposure. Consequently, even if students improved their conceptual understanding of image faults through the interventions, this knowledge may not have translated into better hands-on performance. Unlike the PowerPoint task where errors are immediately visible and correctable, the radiographic task provides delayed and less transparent feedback, potentially limiting the extent to which EMT-related learning could be expressed behaviorally within a single session.

The current study is considerably comparable to the study by Gardner and Rich [5], in which they showed that vicarious EMT (analyzing an error-laden demonstration video) improved immediate simulation performance for radiographer students compared to viewing only correct procedures, with higher blinded ratings and lower variability after a single case-based session. Both their conventional and EMT groups of students reached a mean performance score of >9 (out of 10). However, their between-group difference was statistically significant. While Gardner and Rich placed learners in a dynamic patient scenario requiring adaptation (pain, mobility constraints), our phantom-head context may have minimized adaptive problem-solving demands at test compared to real patient settings, lacking issues such as gag reflex, limited opening, pain on biting the X-ray holder, and missing teeth. Meanwhile, EMT has also been shown to improve the adaptive expertise of medical residents in learning head computed tomography interpretation [11], which was not investigated in the current study focusing on image acquisition.

The current study findings may initially appear counterintuitive. In particular, both groups demonstrated improvements in knowledge and confidence following the intervention, while phantom-head performance showed minimal change. This apparent dissociation between cognitive and psychomotor outcomes suggests that increases in conceptual understanding do not necessarily translate into immediate improvements in technical performance. Radiographic image acquisition is a complex psychomotor task requiring spatial positioning, manual dexterity, and real-time judgment. While knowledge-based understanding of image faults can be acquired relatively quickly, the transfer of this knowledge into procedural execution likely requires repeated deliberate practice over time. From an educational perspective, these findings suggest that short interventions may be effective in improving conceptual understanding but insufficient on their own to enhance hands-on technical competence. More extended training, repeated practice opportunities, and exposure to varied or challenging scenarios may be required to facilitate meaningful improvements in procedural skills. Similarly, a prior study investigated if students would learn skull bone anatomy better with the provision of human dry skulls during the lecture [12]. It was concluded that the students’ post-intervention knowledge scores were comparable regardless of the presence or absence of the skulls. Here, several design features in our study could have potentially attenuated between-group differences. The foremost reason could be ceiling effects and task alignment. Students have already achieved high baseline performance on phantom-head imaging (>90% acceptable/excellent), leaving limited potential for either pedagogy to demonstrate superiority within one session. Moreover, our post-intervention tasks were highly similar to the pre-intervention set (same phantom, standard holders, repeatable projections), aligning more with analogical transfer than adaptive transfer, the very scenario in which EMT did not typically outperform stepwise instruction [1,13]. Results might be different if our post-intervention tasks used different materials, such as using a different set of MCQs and using X-ray holders from a different brand.

Dose and timing of EMT exposure may also matter. EMT benefits in prior work often was seen from richer or repeated exposure with opportunities to monitor, evaluate, and encourage after errors and failure [13]. Unlike making PowerPoint slides, a single 15 min “deliberate erring” block may be insufficient to produce measurable gains in dental radiographic taking within the same session. Brief inductions may not be adequate to shift durable beliefs or behaviors, whereas more concrete prompts and longer interventions may produce clearer downstream effects [14]. More importantly, the findings should not be interpreted as suggesting that additional practice is unnecessary. Rather, they highlight that short, isolated interventions, whether traditional or error-based, may be insufficient to alter technical performance when baseline competence is already high.

A strength of this study is that it is one of the first randomized tests of an error-based pedagogy in dental radiography using both knowledge and hands-on outcomes with a sizable cohort and standardized equipment. We also used phantom-head performance rather than vignette ratings alone, aligning with the notion in medical education to test error-related learning in simulated practice before translating to patients [2].

This study also has some limitations. First, the short EMT dose and immediate outcomes have likely understated EMT potential. A delayed practical assessment could determine whether the intervention resulted in sustained improvements in radiographic performance and the extent of knowledge retention. Second, we did not measure the self-regulatory mediators (metacognition, emotion control) that explain EMT gains, thereby limiting mechanistic inference. In addition, all radiographs were assessed by a single calibrated examiner using predefined criteria. While this ensured consistency, inter-examiner reliability was not evaluated. Meanwhile, students were allowed to repeat faulty images, but only the initial attempts were analyzed, and retake frequency was not recorded. Consequently, potential differences in retake behavior between groups could not be assessed. This is relevant because repeated attempts may influence skill acquisition and could have influenced the comparability of image quality outcomes between groups. Moreover, we only assessed the within-session effect and not the long-term effect of the intervention due to a lack of class time. Even though randomization was performed at the small-group level (4–6 students per group), for pragmatic reasons and given the educational setting, analyses were conducted at the individual level. We acknowledge that this approach may underestimate variance due to clustering effects and therefore may result in conservative interpretation of between-group comparisons. Given that no statistically significant differences were observed, the risk of false-positive findings due to clustering is minimal. Another limitation relates to the use of a 10-item MCQ test with identical questions administered before and after the intervention. While this approach allowed direct comparison of responses, it may have introduced a testing effect, whereby students improved scores through recall rather than true conceptual understanding. As internal consistency of the MCQ was not formally assessed (e.g., using KR-20) and parallel forms were not employed, the magnitude of this potential bias cannot be determined. Therefore, improvements in MCQ scores should be interpreted cautiously.

The results showed that EMT holds promise of being at least as good as traditional methods; therefore, a longer study could be done to further evaluate the possibilities of its long-term effects or its applicability in other dental disciplines in the future. To determine if there will be any significant between-group differences, the clinical performance of students on real adult patients should be subsequently tracked as the actual anatomical constraints will be very different from using a phantom head. Another direction for future studies is to use virtual reality (VR) simulation-based training, as recent studies have shown that the use of such VR simulation training could effectively improve students’ ability to detect and prevent panoramic positioning errors without involving real radiation [15,16]. Preliminary results from an augmented reality (AR) study also showed that students feel positive about using an AR application to learn periapical radiographic techniques [17].

## 5. Conclusions

Within the limitations of this study, a single session of deliberate error-based learning did not produce measurable advantages over conventional teaching in a phantom-head radiography setting. Nonetheless, the study provides preliminary evidence on the feasibility of implementing EMT-inspired strategies in dental education and highlights important contextual factors, such as feedback visibility, task structure, and baseline performance, that may influence their effectiveness. These findings offer useful guidance for the design of future EMT-based interventions in dental radiography training.

## Figures and Tables

**Table 1:** Group data regarding MCQ and phantom-head scores (mean ± SD).

Data	Conventional Group (n = 45)	EMT Group (n = 42)	ICC	Design Effect
Pre-MCQ score (0–10)	7.56 ± 1.55	8.00 ± 1.38	0.25	1.83
Pre-MCQ confidence (1–5)	2.29 ± 0.94	2.52 ± 1.25	0.08	1.27
Post-MCQ score (0–10)	9.58 ± 0.72	9.67 ± 0.53	0.08	1.26
Post-MCQ confidence (1–5)	4.00 ± 1.09	4.26 ± 0.94	0.15	1.49
Pre-phantom-head score (% of max)	72.9 ± 19.3	76.4 ± 15.3	0.01	1.03
Post-phantom-head score (% of max)	75.0 ± 18.2	76.8 ± 15.2	0.00	1.00

All data showed no significant group differences (*p* > 0.05).

**Table 2:** Breakdown of radiographs in terms of diagnostic image quality.

Image Quality	Pre-Intervention		Post-Intervention	
	Conventional Group	EMT Group	Conventional Group	EMT Group
Unacceptable	22 (9.7%)	15 (7.4%)	11 (6.8%)	11 (7.7%)
Acceptable	75 (33.0%)	61 (29.9%)	50 (31.1%)	48 (33.6%)
Excellent	130 (57.3%)	128 (62.7%)	100 (62.1%)	84 (58.7%)

The numbers are numbers of radiographs graded.

## Data Availability

The original contributions presented in this study are included in the article. Further inquiries can be directed to the corresponding author.

## Abbreviations

The following abbreviations are used in this manuscript:

EMT	Error management training
MCQ	Multiple-choice question
VR	Virtual reality
